# Novel, disposable, self-inserted, vaginal device for the non-surgical management of pelvic organ prolapse: efficacy, safety, and quality of life

**DOI:** 10.1186/s12905-022-02057-6

**Published:** 2022-11-18

**Authors:** Elan Ziv, Tsvia Erlich

**Affiliations:** ConTIPI Medical Ltd., 2 Alon Hatavor st., 3088900 Caesarea, Israel

**Keywords:** Pelvic organ prolapse, Non-surgical management, Disposable vaginal device, Self-inserted device

## Abstract

**Background:**

We evaluated a novel disposable, collapsible, ring-shaped vaginal device that is self-inserted within an applicator and removed with a string. The device was developed to overcome the drawbacks of existing ring pessaries for non-surgical pelvic organ prolapse management (POP).

**Methods:**

The primary objective efficacy endpoint of this prospective, interventional, multicenter, self-controlled, and home-use study was the proportion of subjects with improved staging on the Pelvic Organ Prolapse Quantification (POP-Q) scale. Subjective efficacy was assessed using the POP symptoms alleviation score. Safety was evaluated by recording the rate and incidence of adverse events (AEs) in a daily diary, and quality of life (QoL) was evaluated using the modified Pelvic Floor Impact (PFIQ-7) and Pelvic Floor Disability Index (PFDI-20) questionnaires.

**Results:**

A total of 94 usage cycles were observed in a group of 52 participants (mean age 60.2 ± 10.5 years, 81.1% postmenopausal) who used the device for 3558 days. Of these, 24 participants completed one usage cycle, 14 completed two usage cycles, and 14 completed three usage cycles with 28–45 days of ProVate use in each usage cycle. All patients experienced greater than two POP-Q stage reductions. The descent was completely reduced to POP-Q stage 0 in 97.8% of participants. The POP symptom alleviation questionnaire showed significant subjective efficacy (*P* < 0.0001). The modified PFDI-20 and PFIQ-7 scores also improved substantially (*P* < 0.0001 for both). There were 91 nonserious device-related AEs: 98.9% were mild and 87.9% anticipated, with no vaginal infection, and one case of urinary tract infection.

**Conclusion:**

The novel device substantially reduces prolapse and provides significant subjective POP symptom relief and QoL improvement, with minimal AEs. The device may enable women to self-manage their prolapse with a small, disposable device that minimizes self-touching and frequent dependency on the clinic.

**Trial registration:**

Clinical.Trials.gov, NCT02239133, posted September 12, 2014 (retrospectively registered).

## Background

Although minor degrees of pelvic organ prolapse (POP) affect up to 75% of women who have delivered vaginally [1], symptomatic POP affects 3–8% of the female population [2–4]. Approximately 3.5 million women in the United States seek medical assistance for symptomatic POP [5], with 210,000–300,000 undergoing surgical treatments per year and the rest being managed with vaginal pessaries, mainly ring-shaped, or remaining untreated. Although data on the proportion of pessary users is scarce, a 1:1 ratio of pessary users to women with untreated POP can be assumed [6]. Pessaries have been used in the non-surgical management of POP for decades; they are considered effective and safe. However, existing pessaries have substantial drawbacks, which limit their widespread use [7]. Moreover, they are associated with a high rate of discontinuation, which exceeds 50% within 12 months [8], with the major reasons being failure to retain the pessary and inability to insert and remove the device by the user, desire for another treatment modality (e.g., surgery), adverse events (AEs), and sexual disturbances. Existing ring pessaries are reusable only [9] and are large and intrusive, with diameters ranging from 54 to 110 mm. They are partially squeezed (reduced dimensions) during insertion but fully opened during removal, resulting in the most common AE-discomfort and pain when pressing and widening the introitus.

We assumed that more women than previously believed require or want non-surgical POP management, and that vaginal pessaries may be a viable option for them. However, they are hesitant to use them due to multiple difficulties, just as many healthcare providers (HCPs) are hesitant to recommend a treatment with so many complications [10]. The huge gap between existing cumbersome pessary management and women’s desire for a more pleasant, self-manageable, and comfortable POP control, including unhindered intercourse, necessitated the development of a new device that combined the benefits of a ring pessary with substantial reductions in, or elimination of, its major drawbacks. To develop a new treatment option, we conducted a literature search and HCPs surveys for understanding the most bothersome complaints related to existing ring pessaries and designed mitigation options to overcome each complaint (Table 1). Following this, we developed the ProVate device, which has a slender (28 mm) body, that transforms into a ring pessary with six sizes (61–91 mm) and is intuitively self-inserted/removed, similar to how a menstrual tampon is inserted and removed.

**Table 1:** Identified downsides of existing ring pessaries and possible mitigations

Identified downsides	Existing ring pessaries	Mitigation and designing of a new POP device
Discomfort/pain during insertion, usage, and removal	Hard, durable large bodies (Diameter of 54–110 mm). Device expands at the level of the introitus, causing pain/discomfort. Partially squeezed during insertion but full-sized during removal.	A softer and pliable device when fully deployed. Insertion and removal in small dimensions within an applicator. Expansion to intended size only when already within the vagina.
Usability cycle	A reusable only device. Needs cleaning after removal and before next insertion. Biofilms accumulate when in use for many months/years	A disposable only device. No need for handling and cleaning. No biofilms: a new device at every insertion.
Insertion	Manual insertion only: either by a healthcare provider or by the user in the small amount of women who can self-care.	Insertion by the user herself, within a disposable applicator, at her will, regardless of place and time.
Removal	Manual removal only in the large dimensions: either by a healthcare provider, or by the user in the small amount of women who can self-care	Removal by the user herself, by pulling a string at her will, regardless of place and time. The device collapses to small dimensions for comfortable removal.
Length of use	Either every 3+ month by a healthcare provider, or every 1–7 days in the small amount of cases where the user is able to self-care.	Each device should be used for a limited length of time, preferably up to a week.
Adverse events	Vaginal discharge, irritation, infection and foul smell are well described known adverse events.	A disposable only device, with short usage duration, is expected to substantially reduce or eliminate adverse events.
Dependency on the clinic	A reusable device which is re-used for years, requires, in most cases, dependency upon clinic visits.	No dependency upon clinic visits. Device is self-inserted, Irrespective of time and place.
Other points	Need to self-touch when able to self-care. Removal before intercourse is available only in women who can self-care.	No need of self-touch. Device comes ready for use within a disposable applicator. Easy removal before intercourse.

Identified problems with currently available ring pessaries which cause women to refrain from using them or to discontinue usage, as well as possible mitigation options

**Fig. 1:**
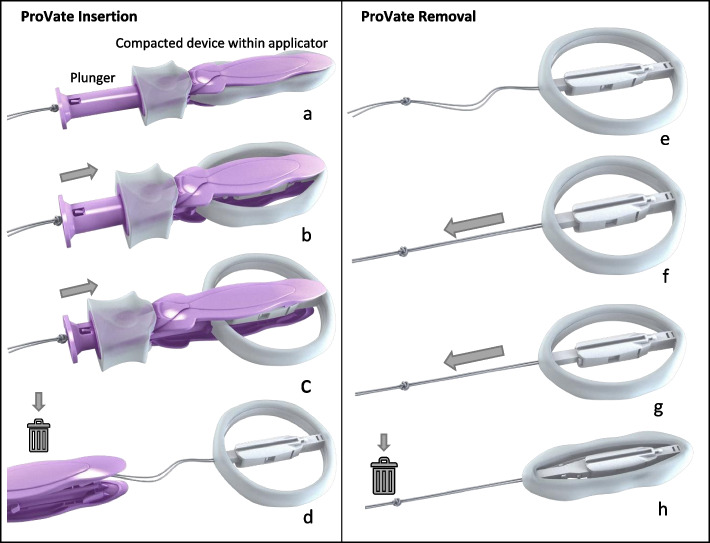
The ProVate Device with its various shapes during insertion and removal. The ProVate device is provided clean and individually wrapped. It is available for immediate vaginal insertion using a disposable applicator (**a**). During vaginal insertion, which is similar to inserting a menstrual tampon, the plunger is pushed, and the slender compacted device within the applicator gradually enlarges to become a ring (**b**). After fully pushing the plunger, the ring becomes fully deployed (**c**). The applicator then separates from the ring and is removed from the vagina for disposal, leaving the string available for later removal (**d**). The deployed ring may remain in the vagina for up to 7 days (**e**). A pull on the string collapses the ring into is its slender pre-insertion size for comfortable removal and disposal (**f**–**h**). (Source: ConTIPI Medical Ltd., with permission)

ProVate (Fig. 1) is a small, disposable, flexible, and self-expanding vaginal ring pessary that comes ready for use within an applicator and with easy removal after a collapse by pulling a string. Like existing rigid ring pessaries, ProVate was designed to function as a scaffold that lifts the prolapsed vaginal walls once in place. Expectedly, when pessaries of equal size are used, their intravaginal mechanical function should be equivalent. Furthermore, their objective and subjective efficacies and impact on the quality of life (QoL) are expected to be similar. However, with ProVate, fewer AEs as well as enhanced user experience and satisfaction are expected, allowing women to self-manage POP and their intimate behavior [11].

The design of a prospective longitudinal home trial to assess objective and subjective efficacies, safety, QoL, and users’ satisfaction was driven by the comparable functionality of ProVate and a ring pessary. The following were the trial’s specific characteristics:

- A single-arm trial in which each user served as her own control (demonstrating quantifiable POP comparisons before and during using the device through well-established performance indicators, such as the Pelvic Organ Prolapse (POP-Q) quantification scale [12] and validated QoL questionnaires [13]).
- A longitudinal, hypothesis-driven, and statistically powered study where some participants used the device for more than one ~45-day usage cycle (e.g. 2–3 usage cycles). This allowed data collection from 94 usage cycles of ~45 days each and from users who repeatedly used the device during 2 & 3 consecutive usage periods for over 2 years. This design facilitated follow-up of users during longer periods, over long time span, with a larger number of evaluable participants (94 usage cycles in 52 partcipants).

The study assumed that although use was moved into the homes and hands of laymen, the device’s efficacy (prolapse stage and related symptoms) in reducing POP would remain high. It was also anticipated that AEs would remain low after insertion and removal in small dimensions of an easy-to-use disposable device for a short period of time. Women can replace the device as frequently as they wish, with limitations of up to 7 days per device, to ensure the low rate of AEs, as this complies with the guidelines of the Society of Obstetricians and Gynecologists of Canada for women who can comply with pessary self-care [14].

## Methods

This research was a prospective, interventional, multicenter, one-arm, open-label, self-controlled, home-use study. The study’s objective was to confirm the efficacy and safety of the ProVate device for regular use. The study was performed following the ethical standards in the Declaration of Helsinki. Furthermore, the institution’s ethics committee approved the research (Assuta-Maccabi Helsinki committee, app #2014038), after which each participant provided written informed consent.

The study was conducted in three outpatient gynecology/urogynecology clinics in Israel between August 2014 and June 2016. Symptomatic participants were recruited from the clinics’ database or following advertisements.

**Fig. 2:**
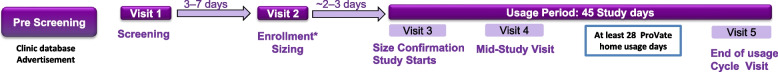
Flow diagram of methodology comparing POP- Q results from the end of visit 5 with those of baseline

Figure 2 presents the study flow and device usage. After screening (visit 1), eligible participants were fitted with the correct size (visit 2) and underwent training on device use. Size confirmation was conducted at the study clinic following 2–3 days of home use (visit 3). Then, the subject was refitted if the size was either too small (causing expulsion) or too large (discomforting). During the usage period, participants were instructed to use as many devices as they wished for 1 to 7 days each, and to fill in a daily diary, documenting each device’s length of use, functionality, and AEs. The home-use portion of the study (termed the *usage period* or *usage cycle*) began after visit 3 and lasted up to 45 days. Participants were instructed to use ProVate for at least 28 days within the 45 days. An ultrasound scan was conducted to estimate the post-void residual (PVR) urine before and while using ProVate. Participants were examined vaginally during each clinic visit by the same gynecologist/urogynecologist to assess the stage of prolapse (POP-Q scale) with or without the device and look for signs of infection, bleeding, and vaginal wall trauma.

Similar ProVate models were tested in an iterative, consecutive fashion, including the final marketed version. Changes between device models were limited to the applicator, whereas the actual ProVate ring, which affects efficacy and safety, remained the same.

Inclusion criteria included women aged 21–80 years with symptomatic sensation of vaginal prolapse, diagnosed with POP-Q stage 2–4 prolapse in one or more sites along the vaginal walls, ability to use both hands and insert a device into the vagina, and the ability to retain a 61- to 91-mm pessary. Exclusion criteria included previous inability to accommodate tampons or vaginal pessaries; current participation in another clinical study; comorbid condition(s) or severe systemic diseases that could limit the subject’s ability to participate in the study; pregnancy, suspected pregnancy, or intention to become pregnant during the study; abnormal vaginal bleeding in the previous 6 months; previous vaginal surgery during the preceding 3 months; severely atrophic vagina; existing vaginal or vulvar laceration; symptomatic vaginal or urinary tract infection, as determined by physical examination and lab results; recurrent urinary tract infections; and abnormal cervical cytology.

The primary endpoint of this study was the proportion of participants who showed an improvement of at least 1 stage from baseline on the POP-Q scale on the fifth visit while using ProVate, as evaluated by per-protocol (PP) analysis. The analysis tested the null hypothesis that the proportion of participants with an improvement from baseline was <70%. The alternative hypothesis was that the proportion of participants with an improvement was ≥70%. The null hypothesis was tested using the exact binomial test. The secondary objective efficacy endpoint was the proportion of participants who were eventually evaluated as POP-Q stage 0 or 1 prolapse on the final visit. The secondary subjective endpoints included the improvement of POP symptoms (assessed by an author-compiled POP symptoms alleviation questionnaire), improvement in QoL (assessed using the modified PFDI-20 and modified PFIQ-7 questionnaires), and participant satisfaction with the device (evaluated by an author-compiled questionnaire).

Objective efficacy, or improvement in the prolapsed stage, was evaluated at all study visits using the POP-Q scale. Additionally, subjective efficacy was assessed using the POP symptoms alleviation score, which was developed and compiled by the authors to assess the change in POP-related complaints before and during treatment. Ten specific POP-related complaints were graded on a scale ranging from 0 to 4 (0 = *no complaint at all* to 4 = *significant complaint*), and scores from visit 1 (before using the ProVate) and visit 5 (while using the ProVate) were normalized to the 0–100 scale, analyzed, and compared.

Changes in QoL were assessed using the applicable parts of the validated PFDI-20 and PFIQ-7 QoL questionnaires (i.e., only those questions that are pertinent to POP). In the modified PFIQ-20 score, 10 of the 20 questions in the original questionnaire were used to assess the burden of pelvic floor disorders due to specific inabilities. Possible scores range from 0 to 4, where 0 = *not at all* and 4 = *very much*. In the modified PFIQ-7 questionnaire, which quantifies the burden of various pelvic floor disorders on the ability to perform certain daily activities, only the seven questions related to the vagina or pelvis were incorporated, with possible scores ranging from 0 to 3, where 0 = *not at all* and 3 = *very much*. Results were normalized to a scale of 0–100.

Participants were questioned regarding their satisfaction while using the device during the three specific usage steps: insertion, usage, and removal. At the end of the study (visit 5), complaints particular to the use of the ProVate, with emphasis on the ability to insert and remove the device (to exclude hand movement limitation), were recorded in an author-compiled questionnaire. Participants’ responses were recorded on a scale ranging from 0 to 4, where 0 means no complaints (*not having any complaints at all*) and 4 means a high level of complaints. Results were then analyzed and plotted on a scale of 0–100, where 0 indicates *total dissatisfaction from device usage* and 100 means *complete satisfaction*. McNemar’s tests were used to assess the proportion of participants who scored specific questions as *having no complaint at all* (0), before and while using the device, for each item separately.

Safety was assessed by recording the rate and incidence of anticipated AEs, including vaginal wall trauma (e.g., erosions, abrasions, ulcerations), vaginal/urine infections, pain, spotting, discomfort, de novo or worsening urinary incontinence and constipation, rate and incidence of serious AEs, and rate and incidence of all AEs (anticipated and non-anticipated, serious and nonserious, related and unrelated to the study device). AEs were assessed using one of the following methods: daily diary, scheduled meeting with the investigator, nonscheduled call from the subject, and scheduled weekly telephone call to the subject.

The full analysis (FA) set included all eligible participants who used at least one device (even if the insertion process was never completed). The FA set served as the principal analysis set for the safety assessment. The PP analysis set included all participants from the FA set who used the study device models for at least 20 days, with no significant protocol deviation. The PP analysis set served as the principal analysis set for the analyses of the primary and secondary endpoints.

Statistical analyses were performed using SAS v9.4 (SAS®, SAS Institute Cary, NC USA) software. Under the assumptions (100% success), the sample size required to test the null hypothesis at a 5% significance level and with 80% power was at least 36 evaluable participants.

## Results

The participants’ mean age was 60.2 ± 10.5 years, and 52.3% were between 61 and 70 years of age; their mean body mass index was 25.48 ± 4.16 kg/m^2^. Of the 151 births reported, 108 were spontaneous vaginal deliveries, 39 were vaginal and required instrumentation, and four required cesarean delivery. The average weight of the newborns was 3662 ± 449 g. Most participants (81.1%) were postmenopausal, with a mean length of amenorrhea of 14.2 years. Moreover, 13 participants used systemic hormone replacement therapy, and 6 used vaginal estrogen cream.

**Fig. 3:**
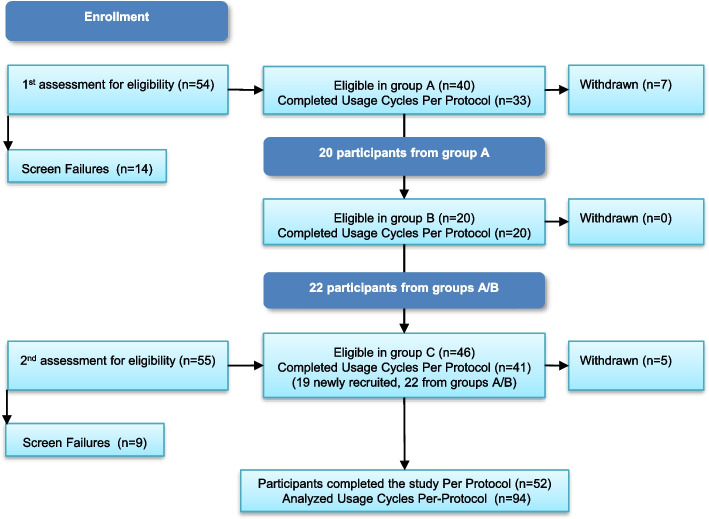
The three phases of the study. Altogether, 94 usage cycles in three clinics were recorded over 2 years with three phases (each phase >6 months apart) Phase A included 33 participants (usage cycles) who completed the study PP, of whom 20 also tested ProVate in phase B. Additional participants were recruited for phase C, which was completed PP by 41 participants of which 22 used the device during phases A/B. Altogether, 94 usage cycles were completed by 52 symptomatic women, of whom 24 completed one usage cycle, 14 completed two usage cycles, and 14 completed three usage cycles. Participants who used more than one model were reconfirmed for inclusion and exclusion criteria before the next usage cycle, which was thus considered as an additional usage cycle

Altogether, 94 usage cycles in three clinics were recorded. This research was a longitudinal study conducted over 2 years with three phases (each phase >6 months apart) that tested slightly different applicators of the device (Fig. 3). No changes to the actual ProVate ring were made. Phase A included 33 participants (usage cycles), of whom 20 were also in phase B. Additional participants were recruited for phase C, which was completed by 41 participants using the final applicator (of these, 22 participants already used the device at phase A/B). Altogether, 52 symptomatic women completed this study; of these, 24 completed one usage cycle, 14 completed two usage cycles, and 14 completed three usage cycles with 28–45 days of ProVate use in each usage cycle. Participants who used more than one model were reconfirmed for inclusion and exclusion criteria before the next usage cycle, which was thus considered as an additional usage cycle (altogether: 94).

Eighty-seven new participants were screened, of whom 18 were initial screen failures. Additionally, during the study, 8/69 could not be fitted with available sizes (e.g., wide introitus or short vagina), 3/69 discontinued participation because of AEs, 2/69 were withdrawn because of their inability to insert the device by themselves (short hands and inability to bend back), 1/69 opted for surgery, and 3/69 were removed due to violations of study procedures.

In total, after device sizing and accommodation, during the device usage period only, 992 ProVate devices were used over 3393 usage days in the PP population, with an average of 36.1 ± 5.70 days per subject, and 1592 devices were used over 3558 study days in the FA (safety) group.

### Objective efficacy: Reduction of POP stage

The study population included participants with multiple prolapse sites (e.g., anterior and apical). While using the ProVate device, the POP reduction was not limited to a specific location. Still, upward distension of the vaginal apex resulted in a flattening and decline of the prolapse at other vaginal sites. General efficacy analyses were conducted on the PP set using 94 usage cycles. However, in two cases, the POP-Q results after the study were missing. Hence, the reduction in POP was calculated over only 92 cases.

**Fig. 4:**
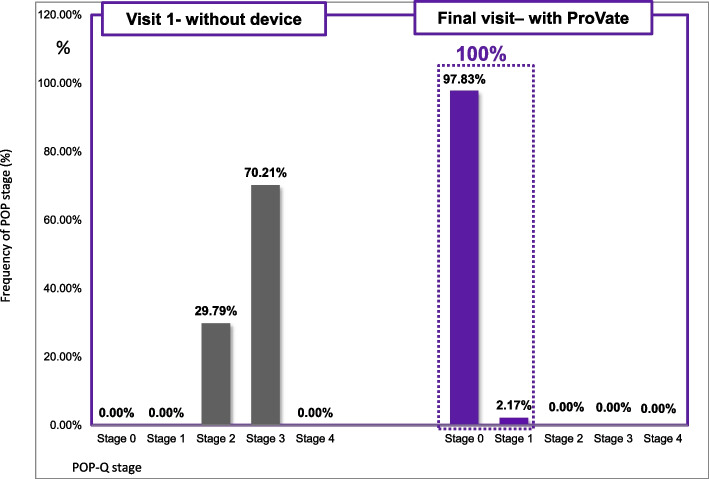
Comparison of objective efficacy (POP-Q staging) before and while using the ProVate Device. Before using ProVate, 70.21% of users had POP-Q stage 3 prolapse, whereas 29.79% had stage 2 prolapse. While using ProVate, 100% of subjects had a substantial reduction of prolapse to POP-Q stage 0/1. (POP-Q = Pelvic Organ Prolapse Quantification)

In the PP set, the pre-study POP-Q staging included 28 usage cycles (29.79%) with stage 2 prolapse and 66 (70.21%) usage cycles with stage 3 prolapse (Fig. 4), all symptomatic. In all usage cycles (100%), a reduction of at least two POP-Q stages while using the ProVate device (95% exact confidence interval [CI] [96.07; 100]) was observed (Table 2). Besides, in 64 of 66 (97%) cases with POP-Q stage 3 prolapse, a reduction of three POP-Q stages (95% exact CI [89.48; 99.63]) was observed. Therefore, it is evident that the first objective efficacy endpoint was met, and the null hypothesis was rejected (*P* < 0.001).

**Table 2:** Prolapse reduction while using the ProVate Device (POP-Q staging, 94 usage cycles, PP set, POP = Pelvic Organ Prolapse, POP-Q = Pelvic Organ Prolapse Quantification)

POP Reduction Results With The ProVate Device (by POP-Q staging)		Results
		92 usage cycles
Primary Objective Efficacy Endpoint Prolapse Reduction (# of stages reduced)	% of subjects with ≥1 stage POP reduction from baseline	100.00%
	% of subjects with ≥2 stages POP reduction from baseline	100.00%
	% of subjects with ≥3 stages POP reduction from baseline^a^	96.97%
Secondary objective Efficacy Endpoint Final POP-Q Stage Achieved With ProVate at visit 5; (% Subjects)	POP-Q stage 0 while using the ProVate	97.83%
	POP-Q stage 1 while using the ProVate	2.17%
	POP-Q stage 2 while using the ProVate	0.00%

^a^Compared with those who had POP-Q stage 3 prolapse

A secondary objective efficacy endpoint relates to the proportion of participants who eventually had either POP-Q stage 0 or 1 prolapse at the end visit while using ProVate. There was no prolapse (POP-Q stage 0) in 90 usage cycles (97.8%), whereas there was POP-Q stage 1 prolapse in two usage cycles (2.2%; Table 2). Collectively, in 92 of 92 cases (100%), prolapse was reduced to either stage 0 or 1 (*P* < 0.0001).

This improvement was demonstrated at all three study clinics with no statistically significant difference among sites; hence, data from all study sites were pooled.

### Subjective efficacy: Reduction of POP symptoms

**Fig. 5:**
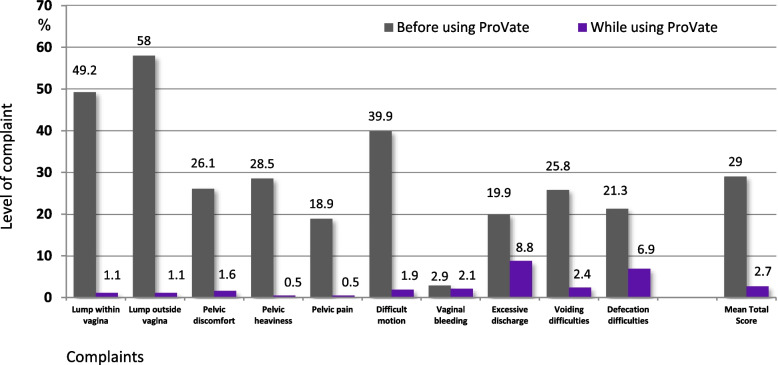
Comparison of complaints before using ProVate and while using ProVate. Complaints were graded 0–4 using the POP alleviation score (0 being “no complaint at all” and 4 being “significant complaint”). Scores on visit 1 (before using the device) and visit 5 (while using the device) were normalized to the 0–100 scale and were analyzed and compared (94 usage cycles, 992 devices, 3393 usage days, PP population; mean total score *p* < 0.0001, POP = Pelvic Organ Prolapse). (Source: ConTIPI Medical Ltd., with permission)

Figure 5 shows results from the author-compiled POP symptoms alleviation scores obtained from visit 1 (before device use) and visit 5 (end visit). The scores were substantially reduced for each of the 10 items, and the mean total score decreased significantly from 29 to 2.7 (*P* < 0.0001).

### QoL questionnaires

**Fig. 6:**
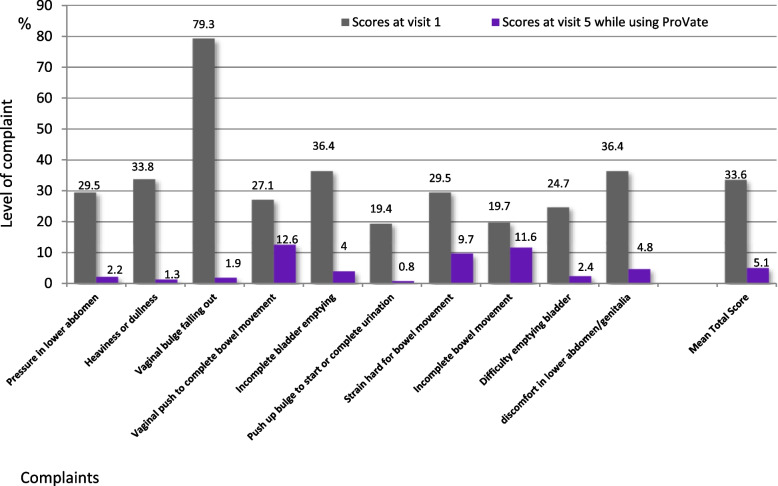
Modified PFDI-20 Quality of life questionnaire. Comparison of POP-relevant questions before using ProVate and while using ProVate. Complaints were graded 0–4 (0 being “no complaint at all” and 4 being “significant complaint”). Scores on visit 1 (before using the device) and visit 5 (while using the device) were normalized to the 0–100 scale and were analyzed and compared (94 usage cycles, 992 devices, 3393 usage days, PP population; mean total score *p* < 0.0001, POP = Pelvic Organ Prolapse, PFDI-20 = Pelvic Floor Disability Index 20)

**Fig. 7:**
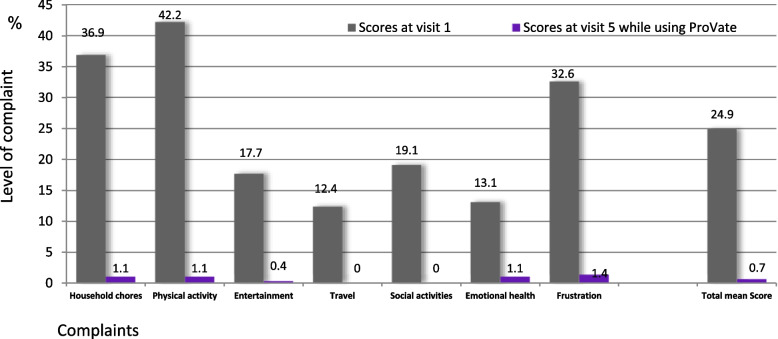
Modified PFIQ-7 Quality of life questionnaire. Comparison of POP-relevant questions before using ProVate and while using ProVate. Responses were graded 0–3 (0 being “no complaint at all” and 3 being “significant complaint”). Scores on visit 1 (before using the device) and visit 5 (while using the device) were normalized to the 0–100 scale and were analyzed and compared (94 usage cycles, 992 devices, 3393 usage days, PP population; mean total score *p* < 0.0001, POP = Pelvic Organ Prolapse, PFIQ-7 = Pelvic Floor Impact Questionnaire, short form 7)

A statistically significant decrease in all items (implying an improvement in QoL regarding POP) was observed on both the modified PFDI-20 and PFIQ-7 QoL questionnaires. Figure 6 shows a substantial decrease in all items of the modified PFDI-20 scores. The mean total score decreased from 33.6 before using ProVate to 5.1 while using ProVate (*P* < 0.0001). The proportion of participants who scored “not at all” for specific items ranged from 5.3 to 76.6% at baseline. Subsequently, this value increased (80.6 to 98.9%) at the study end (*P* < 0.0001). Figure 7 demonstrates a substantial decrease in all modified PFIQ-7 items scores. The mean total score decreased from 24.9 before using ProVate to 0.7 while using ProVate (*P* < 0.0001). The proportion of participants who scored “not at all” for specific items ranged from 33.0 to 81.9% at baseline. However, these values increased to 95.7% at the study end (*P* < 0.0001).

### Subject’s satisfaction score

**Fig. 8:**
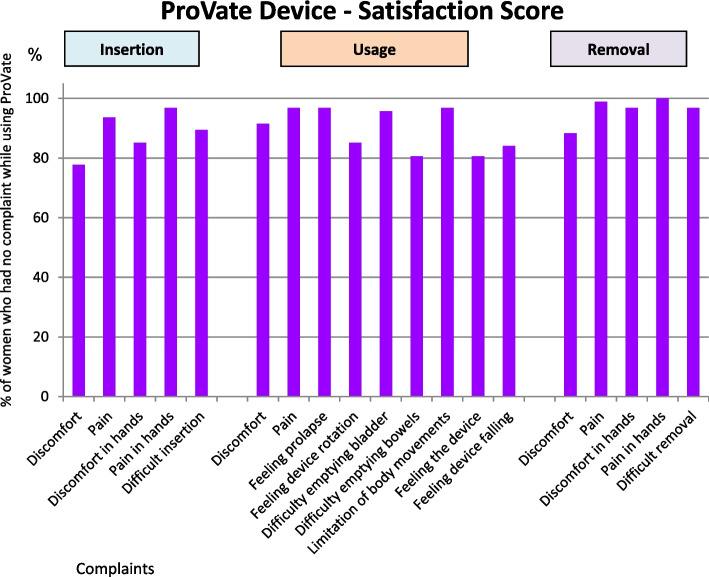
ProVate Satisfaction Score. Chart shows only responses of “no complaints at all” by study end. Responses were graded 0–4 (0 being “no complaint at all” and 4 being “significant complaint”), normalized to the 0–100 scale, analyzed, and compared (94 usage cycles, 992 devices, 3393 usage days, PP population)

As shown in Fig. 8, the responses to the satisfaction questionnaire were highly favorable. This figure shows only the results with scores of “not having any complaints at all” given for all 14 items, ranging from 77.7 to 100%. Most items scored at least 90%.

### AEs

General safety analyses were conducted on the FA set; 98.9% of AEs were mild, and 87.9% were anticipated. There were no device-related serious AEs, and all AEs resolved completely with no sequelae. In all three study parts, while 124 AEs were reported in the FA set, 109 AEs were observed in the PP set. The most common AEs were discomfort and spotting, anticipated for all devices used vaginally during initial usage stages.

Table 3 shows the distribution of device-related AEs within the FA and PP sets. In the FA set, 91 AEs occurred in 51 participants (45.95% of the FA set) while using 1592 devices over 3558 usage days. However, in the PP set, 77 AEs occurred in 44 participants (46.81% of the PP set) while using 992 devices over 3393 usage days.

**Table 3:** Summary of device-related adverse events (AEs) while using ProVate

Adverse Event	FAS (111 confirmed/reconfirmed participants, 51 with device-related AEs)			PP (94 completed usage cycles, 44 with device-related AEs)		
	Number of device-related AEs	Number of participants with device-related AEs	% of device-related AEs within FAS	Number of device-related AEs	Number of participants with device-related AEs	% of device-related AEs within PP
**Anticipated AEs**						
Spotting	26	20	18.02	23	18	19.15
Discomfort	21	16	14.41	16	12	12.77
Pain	9	8	7.21	6	6	6.38
Mild Pain	7	6	5.41	7	6	6.38
Vaginal wall trauma	7	4	3.60	7	4	4.26
De-Novo SUI	2	2	1.80	1	1	1.06
Vaginal irritation	2	2	1.80	2	2	2.13
Substantial discomfort	1	1	0.90	1	1	1.06
Odor	1	1	0.90	1	1	1.06
UTI	1	1	0.90	1	1	1.06
Presumptive UTI	1	1	0.90	1	1	1.06
Local vaginal pressure	1	1	0.90	1	1	1.06
Vaginal pain	1	1	0.90	1	1	1.06
**Non-anticipated AEs**						
Pressure on urinary bladder	2	2	1.80	1	1	1.06
Vaginal burning sensation	2	1	0.90	2	1	1.06
Lower extremities pains	1	1	0.90	.	.	.
Poor urinary stream	1	1	0.90	1	1	1.06
Asymptomatic Bacteriuria	1	1	0.90	1	1	1.06
Difficulty emptying bladder	1	1	0.90	1	1	1.06
Frequent urination	1	1	0.90	1	1	1.06
Vaginal discharge with odor	1	1	0.90	1	1	1.06
Vulvo-vaginal burning	1	1	0.90	1	1	1.06
**Total**	**91**	**51**	**45.95%**	**77**	**44**	**46.81%**

In the Full Analysis Set (FAS), there were 111 confirmed/reconfirmed participants in the 3 phases of the study, some participated in more than one phase, >6 months apart. 51 (45.95%) had 1 or more device-related AEs. Altogether there were 91 device-related adverse events while using 1592 devices over 3558 usage days

In the per-protocol (PP) set, there were 94 completed usage cycles, where in 44 (46.81%) there were 1 or more device-related AEs. Altogether there were 77 device-related adverse events while using 992 devices over 3393 usage days

*SUI* Stress Urinary Incontinence, *UTI* Urinary Tract Infection

Seven cases of vaginal wall trauma (accounting for 7.6% of device-related AEs) were seen at the beginning of the study in only 4 (3.6%, FA set) participants.

The largest proportion of AEs consisted of sporadic AEs, usually one to two complaints each. In the 91 potentially device-related AEs (FA set), 33 (36.3%) were reported during sizing and 58 (63.7%) during the device usage phase. Most of the device-related AEs occurred within 1 week from the study start (58.9%) and during the use of the first five devices (75.8%). This finding shows a typical learning curve for the new product.

### Specific safety points: Vaginal infections, urinary tract infection, and urine retention

There were no signs or symptoms of vaginal infections (based on self-report or vaginal examination). However, one case of urinary tract infection (UTI) was observed, which was treated with antibiotics. A case of presumptive UTI was also observed, in which the physician commenced treatments without a lab test and without reporting to the site.

PVR urine volume was studied by ultrasound scan before insertion of the first device and with the device deployed within the vagina (visit 4). There was no significant difference in PVR between before (15.0 ± 15.56 mL [range, 0–53.5 mL]) and while using the ProVate (14.1 ± 21.9 mL [range, 0–90.7 mL]).

## Discussion

While offering new aspects of home self-insertion and removal of a disposable device, this study aimed to demonstrate the substantial objective and subjective efficacy and safety of ProVate, together with the reported increase in QoL and users’ satisfaction.

The primary objective efficacy endpoint of the study, the proportion of participants with at least a one-stage reduction in the POP-Q stage, was met and shown to be 100%. In >96%, two or more stages on POP-Q were reduced. In >97%, prolapse was reduced to stage 0, regardless of the initial stage. The author-compiled POP symptoms alleviation score showed significant improvement in prolapse symptoms while using the device, reflecting substantial subjective efficacy, regardless of the objective efficacy.

The prolapse reduction from using ring pessaries was associated with increased QoL [15]. In this study, the participants’ QoL improved considerably with ProVate. This finding was reflected in the results from the modified PFIQ-7 and PFDI-20. Furthermore, as indicated by the specific questionnaire, the users’ satisfaction was significant.

Medical literature cites conflicting data on the prevalence of AEs within groups of pessary users. For example, although Hanson et al. [16] reported that only 14.5% of users had any complaints, Bai et al. [17] reported that 73.1% experienced AEs, and Sarma et al. [18] reported that 56% had AEs. This large variability reflects a difference in reporting. Here an ongoing daily/weekly follow-up of complaints, together with a daily diary, will likely lead to a much larger proportion of complaints. However, the rate of AEs was still relatively low with ProVate, and most AEs were minor, mild, and anticipated.

Most anticipated AEs within the study occurred during the sizing phase and at the first week of the usage phase. These issues diminished when participants became more experienced with the use of the device (“learning curve effect”). Discomfort and spotting accompany the initial use of any intravaginal device, including menstrual tampons, mainly in those with estrogen deprivation [19]. As anticipated, these two were the most prevalent AEs upon the initial usage of ProVate. However, their incidence was low and diminished with usage experience.

Vaginal wall trauma, an anticipated AE, occurs in 3–24% of pessary users [20]. In this study, the low rate of vaginal wall trauma (3.6%) is attributable to the user’s inexperience and initial trials of inserting the device. This mechanism of wall trauma is probably different from where trauma is caused by prolonged pressure [21] (“pressure ulcers”) by a reusable pessary.

Vaginal purulent discharge, itching, and foul smell are common among pessary users [22, 23], specifically among those who use them for long periods, perhaps due to biofilm formation [24]. However, no complaints, clinical signs, or symptoms of vaginal infection arose in this study while using ProVate. This finding is attributed to the frequent replacement of a fresh and clean device.

Compared with most other studies that rely on memory recall only, a strength of this study is the daily collection of AEs in a diary of more than 3558 usage days. Additional strengths include the long usage period (3558 usage days) under strict supervision and the design of the study whereby a strict patient follow-up was used, which allowed for the early detection of specific AEs (e.g., vaginal wall trauma) and corrective actions (e.g., instructing participants how to properly insert the device). Another strength of the study is its longitudinal design conducted over 2 years, enabling adequate follow-up on possible late AEs and possible prolonged usage habits.

A limitation of this study is its single-arm design, with data collected during use of the ProVate device only. However, it should be noted that established and validated key performance indicators (POP-Q, PFDI-20, PFIQ-7) were used for the main comparisons to investigate functionality, performance, and usability, and that a comparison with existing ring pessaries was not necessary to establish required data and thus was not included in the current study. Therefore, a future study comparing results between ProVate and existing ring pessaries is needed. Another limitation is the rather short follow-up duration of up to 45 usage days within each usage cycle; however, this is undoubtedly sufficient to demonstrate the efficacy and safety of a new device. Simultaneously, participants who repeated usage within up to three usage cycles in 2 years actually demonstrated prolonged usage habits.

## Conclusion

In an era where many women avoid clinic visits (e.g., during a pandemic) and when avoidance of treatment can lead to complications, ProVate is an example of a simple and available self-use home management method. This new management modality for POP may elevate compliance with POP treatment among untreated patients. However, further studies are needed to learn more about other characteristics of this device.

## Data Availability

The data that support the findings of this study are confidential assets of ConTIPI Medical Ltd. Restrictions apply to the availability of these data, as they are the company’s confidential and restricted data and are therefore not publicly available. However, data may be available from the corresponding author upon reasonable request and with permission of ConTIPI Medical Ltd.

## Abbreviations

AE: Adverse Event
FA: Full analysis
HCP: Healthcare providers
PFDI: Pelvic Floor Disability Index
PFIQ: Pelvic Floor Impact Questionnaire
POP: Pelvic organ prolapse
POSST: Pelvic Organ Support Study
PP: Per-protocol
PVR: Post-void residual
QoL: Quality of life
UTI: Urinary tract infection
